# Biomass in the manufacture of industrial products—the use of proteins and amino acids

**DOI:** 10.1007/s00253-007-0932-x

**Published:** 2007-03-27

**Authors:** Elinor Scott, Francisc Peter, Johan Sanders

**Affiliations:** 1 Department of Valorisation of Plant Production Chains, Wageningen University, P.O. Box 17, 6700 AA Wageningen, The Netherlands; 2 Industrial Chemistry and Environmental Engineering, Politehnica University of Timisoara, Telbisz 6, 300001 Timisoara, Romania

**Keywords:** Cinnamic Acid, Fumaric Acid, Fossil Resource, Isobutyraldehyde, Enzymatic Decarboxylation

## Abstract

The depletion in fossil feedstocks, increasing oil prices, and the ecological problems associated with CO_2_ emissions are forcing the development of alternative resources for energy, transport fuels, and chemicals: the replacement of fossil resources with CO_2_ neutral biomass. Allied with this, the conversion of crude oil products utilizes primary products (ethylene, etc.) and their conversion to either materials or (functional) chemicals with the aid of co-reagents such as ammonia and various process steps to introduce functionalities such as -NH_2_ into the simple structures of the primary products. Conversely, many products found in biomass often contain functionalities. Therefore, it is attractive to exploit this to bypass the use, and preparation of, co-reagents as well as eliminating various process steps by utilizing suitable biomass-based precursors for the production of chemicals. It is the aim of this mini-review to describe the scope of the possibilities to generate current functionalized chemical materials using amino acids from biomass instead of fossil resources, thereby taking advantage of the biomass structure in a more efficient way than solely utilizing biomass for the production of fuels or electricity.

## Introduction

The depletion in fossil feedstocks, increasing oil prices, and the ecological problems associated with CO_2_ emissions are forcing the development of alternative resources for energy, transport fuels, and chemicals: the replacement of fossil resources with CO_2_ neutral biomass.

The use of renewable feedstocks to prepare products has already been widely explored in a previous era when bio-based raw materials and associated technologies were the most readily available. For example, fats and oils are traditional sources for soap production as well as being the raw materials for lubricants, corrosion inhibitors, and the like. Recently, the impetus has been that bio-based raw materials can make a significant contribution to the ecological and economic production of (new) chemical products and materials and can also be thought of as a useful chemical and technological toolbox. For instance, seeking a new bio-based product to substitute traditional (petro)chemical products can be advantageous ecologically, as it reduces the use of fossil raw materials or is economically interesting because the bio-source has a lower price, or it allows the incorporation of a certain naturally occurring chemical structure that offers better functionality.

However, here, new and different products to those derived from fossil resources are prepared. While this offers opportunity for many inventions, the production of petrochemical-based products has led to chiefly optimized production chains in terms of the economics and performance of the product as well as in aspects such as process optimization and environmental impact. Hence, replacement of petrochemical products with bio-based alternatives often requires the whole production chain to be re-optimized. As this frequently involves many different participants, it is often difficult to convince all parties to take part in developments and changes leading to disruption of the production chain and lack of success in launching new products. Conversely, it may prove more beneficial to not introduce new products but prepare existing chemicals using bio-based raw materials. Thus, it is proposed that the use of bio-based raw materials, instead of fossil raw materials, to prepare current (chemical) products should be the initial move towards a bio-based economy.

### The use of biomass as a raw material

Biomass has found use in technical applications. For example, soybean oil is used as an additive for poly(vinyl chloride), while other fatty oils have been used to make derivatives that can be used as surfactants. Even so, the direct application of naturally occurring structure(s) or biomass raw materials in technical materials is more limited if one considers the production of specific chemicals for a particular product.

Effort to produce chemicals with more control over constant quality and performance has been addressed. Mainly, this has focused on the use of carbohydrates as raw materials and use of biotechnology for conversion. For example, the production of lactic acid is well established by both Purac and Cargill, and applications are found in many industries. With the advent of successful (large-scale) fermentation, technology has also been developed for the synthesis for lactide (from lactic acid) to allow synthesis of poly(lactic acid). In the mid-1990s, Cargill Dow started production of NatureWorks®, which is used as a bio-based and biodegradable material. Other monomers include 1,3-propanediol (Biebl et al. 1999), a comonomer in the synthesis of poly(propylene terephthalate) (Sorona®). Biotechnological preparation of 1,3-propanediol (from glycerol) has been known for some time, but in recent years, developments in metabolic engineering by DuPont and Genencor, allowing glucose to be used as a feedstock, has led to successful commercial production. These technologies focus on “new” types of chemicals and materials with new or specific properties and do not examine the use of biomass as an alternative for the production of chemicals traditionally obtained from the petrochemical industry. The (long) time scales involved for these types of new developments and products relate to not only new products and markets needing to be developed but also to new properties and technologies as well.

The petrochemical industry utilizes simple hydrocarbons such as ethylene to prepare (chemical) products and materials. Here, various process steps together with the use of co-reagents, catalysts, and other processing aids are used to convert the simple structures to more functionalized materials. The production of ethylene from biomass-derived ethanol is known. Using this approach, bio-based alternatives to fossil-derived hydrocarbons are generated; however, it still requires the use of reagents, process steps, energy, and hence, fossil resources to convert this to more functionalized products. It is already known that lower raw material costs and capital investment costs are incurred in the production of nonfunctionalized chemicals compared to more functionalized compounds (Lange 2001). Thus, more efficient use of the functionality of biomass may be exploited to make functionalized chemicals.

It is anticipated that the level of substitution of petrochemical transportation fuels with biofuels will rise to 10% in the coming years. This means that a rise in production of biodiesel from rape and soya will lead to large and excess quantities of glycerol as a rest stream. Indeed, some companies are already investigating the use of glycerol to produce existing chemicals. Solvay has announced it will produce epichlorohydrin from glycerol, using the Epicerol™ process (Solvay Chemicals 2006). Other chemicals from glycerol include methanol and propylene and ethylene glycols. Bio-Methanol Chemie Nederland uses glycerol to produce synthesis gas, which is reformed to make methanol (Chemie Magazine 2006), while Archer Daniels Midland plan to use carbohydrates and/or glycerol as a feedstock for the production of propylene and ethylene glycols (Chemical Week 2005).

While the awareness of large volumes of glycerol from biofuel production is apparent, one should not overlook other waste streams. Indeed, from biofuel production, an immense concomitant waste stream of protein will also be generated. Some of the amino acids present in such proteins could be very suitable raw materials for preparing (highly) functionalized chemicals traditionally prepared by the petrochemical industry. It is the aim of this mini-review to describe the scope of the possibilities to generate current functionalized chemicals using amino acids from biomass instead of fossil resources. While it is intended to show the possibilities, it is also the intention that production of current products could be more attractive to current methods, as functionalized biomass components may offer the possibility to bypass process steps.

## The use of amino acids

The literature for the use and transformation of amino acids is extremely diverse. In general, however, these focus on use of amino acids in nutrition, medicine, or impact on physiological function. The common characteristics of amino acids are the presence of carboxylic acid and amine functionalities, and indeed, most of the (bio-) chemical information is focused in the reactivity and transformations of these groups. For example, the decarboxylation of α-amino acids is well reported in the literature, where in most cases, both chemical and enzymatic decarboxylations can be effected. Where enzymatic decarboxylation is carried out, amino acid specific decarboxylase enzymes are utilized.

In this study, it is intended to illustrate and highlight reactions involving a range of amino acids, where the impetus is the overlap of the end products of these reactions with chemicals currently produced in the petrochemical industry. Table 1 shows some of the chemicals that are currently available and have been shown to be products of the reactions of amino acids.

**Table 1:** Some petrochemical products (current volumes, value, and applications)—could these be made from amino acids?

Traditional chemical product	Current end product volume (tonnes per annum)	Current value (€ per tonne)	Applications
1,2-Ethanediamine	Estimated >1 × 10^6^ tonnes (WW)	1,600–1,750^a^	Rubber chemicals, pharmaceuticals, EDTA synthesis
1,4-Butanediamine	Estimated 10,000s (E)	Estimated >1,600	Nylon-4,6
4-Vinylphenol	Low	–	Poly(vinylphenol) (PVP) for use in electronic devices
Acrylamide	About 0.3–0.5 × 10^6^ (WW)^b^	About 3,000^c^	Polyacrylamide (for water treatment)
Acrylic acid (and esters)	About 2.5 × 10^6^ (WW)^b^	1,450–1,600^a^	
Allylamine	Low	–	Antifungal preparations
Aminoethanol	About 0.5× × 10^6^ (WW)^b,d^	1,250^a^	Detergents, ethyleneamines, purification
Benzaldehyde	–	–	Fragrances
Benzoic acid	About 0.1–0.5 × 10^6^ (WW)^e, b^	About 1050^e^	Dyes, rubbers, preservatives
Catechol	About 20 000 (WW)	About 3800	Photochemicals, printing
Cinnamic acid	Low	–	Flavor and fragrances
Ethylamine	High volume intermediate, low–volume end product	–	Dye intermediate
Fumaric acid	Low-volume end product 50,000 (WW)^f^	About 1,100^f^	Food, resins, paper sizing
Isobutyraldehyde	Estimated > 0.1 × 10^6^ (WW)	–	
Isoprene	About 0.3 × 10^6^ (US)^g^	–	Rubbers
Isopropanolamine	Estimated 10,000s	–	Surfactants, pigments, corrosion inhibitors, and lubricants
Maleic anhydride^h^	About 0.8 × 10^6^ (WW)^b^	1,400–1,600^a^	Unsaturated polyesters, pesticides, lubricant additives
Oxalic acid	Medium	–	Purifying agent in pharmaceutical industry, bleaching agent in textile and pulp industries, rust-remover, wastewater treatment
Propionic acid	About 0.1–0.2 × 10^6^ (WW)^I^	About 850^I^	Antifungal preparations, intermediate in pesticides and pharmaceuticals
Pyrrolidine	Estimated about 10–20,000 (WW)^j^	Estimated >2,000	Pharma
Styrene	5 × 10^6^(US)^g^	1,000–1,100^a^	Plastics
	About 17 × 10^6^(WW)^b^		
Toluene	3 × 10^6^(US)^g^	About 700^a^	Solvent, intermediate to benzoic acid, phenol, stilbene
Urea	5.2 × 10^6^ (E)^j^	210–230^a^	Fertilizers, melamine synthesis
	7 × 10^6^ (US)^g^		
Vinyl pyrrolidone	About 10–20,000 (WW)^b^	Estimated >2,000	PVP
γ-Butyrolactam	About 10-20,000 (WW)^b^	Estimated >2,000	Vinyl pyrrolidine synthesis
ɛ-Caprolactam	About 0.9 × 10^6^ (E)^j^	1,700^a^	Nylon-6
	About 0.7 × 10^6^ (US)^g^		

*WW* Worldwide, *US* United States of America, *E* Europe, *EDTA*, ethylenediaminetetraacetic acid

^a^ICIS pricing 2006

^b^Weissermel and Arpe (1993)

^c^ http://www.the-innovation-group.com (price from 2001)

^d^Production volume exceeds this value as produced as an intermediate.

^e^ http://www.the-innovation-group.com (price from 2000)

^f^ http://www.the-innovation-group.com (price from 1999)

^g^Brown (2003)

^h^Maleic anhydride can be converted to maleic acid. Limited info is known about direct acid synthesis.

^i^CMR 2006

^j^Personal communications

### Alanine

Thermochemical decarboxylation of a number of α-amino carboxylic acids using aromatic aldehydes has been reported (Dose 1957). To prevent the reaction of resultant amines with reactants, *o*- or *p*-substituted aldehydes were used. In the case of alanine, ethylamine was formed. The enzymatic decarboxylation of alanine, resulting in the formation of ethylamine, has also been demonstrated in plants (Crocomo and Fowden 1970). Ethylamine is a high production volume chemical product and is used as an intermediate in a number of applications such as the dye industry.

### Asparagine and aspartic acid

A number of articles are known about the formation of acrylamide in food products and impact on nutrition and toxicity (Taeymans et al. 2004; Arisseto and Toledo 2006; Goekman and Senyuva 2006; Robert et al. 2004). Here, the emphasis is the heat treatment of food products, where the Maillard reaction and, in particular, the presence of asparagine gives rise to the formation of acrylamide. In one report, the formation of acrylic acid from an aspartic acid, β-alanine, and carosine pathway was observed (Yaylayan et al. 2005). Conventional (industrial) synthesis of acrylamide involves the partial acid hydrolysis of acrylonitrile, although recent developments are investigating acid-free systems using Raney copper as well as the use of biotechnology using *nitrile hydratase* in the conversion of acrylonitrile to acrylamide (Chem Systems Reports 2002). The major applications of acrylamide is the manufacture of poly(acrylamide), which is mainly used in the treatment of wastewater. Acrylic acid is commercially produced by a number of routes. Processes based on the use of ethylene oxide and HCN; the carbonylation of acetylene; the hydrolysis of acrylonitrile and the catalytic oxidation of propene are reported.

Other transformations of aspartic acid include the α-deamination of the aspartic acid to produce fumaric/maleic acid. Conventional thinking would suggest that decarboxylation of an amino acid where an amino group is present in the α-position should be principal reaction. However, the susceptibility of deamination/decarboxylation can be different depending on the substrate. In the case of aspartic acid, α-deamination is predominant. One method to obtain the α-deamination of aspartic acid is by hydrothermolysis (Sato et al. 2004). Some studies show that aspartic acid is stable in the temperature range 110–150°C and begins to evolve ammonia at temperatures above 150°C. When reaction takes place for 2 h at 180°C, >50% α-deamination occurs resulting in the formation of fumaric and maleic acid (Sohn and Ho 1995). However, α-decarboxylation of aspartic acid to β-alanine, is also known. Here, aspartate 1-decarboxylase (E.C. 4.1.1.11) is used to catalyze the reaction (Brenda database, http://www.brenda.uni-koeln.de). A photochemical method has also been reported (Takano et al. 2004). Fumaric acid is used mainly produced by fermentation and used in the food and paper industry. However, due to the unsaturated nature of the diacid, it has also been used in the preparation of unsaturated poly(ester) and alkyd resins. Maleic acid is chemically and structurally very similar to fumaric acid—they are isomers of each other—but is produced and used on a limited basis by industry. Maleic anhydride can be used as a source of maleic acid, and it is the synthesis and applications of the anhydride form that has received most attention. However, it has also been reported that maleic acid (from wastewater form processes) can be converted to the anhydride by dehydration or isomerized to produce fumaric acid. The anhydride is produced by a number of processes based on the oxidation of benzene or butane and used as a monomer in unsaturated poly(esters) and an intermediate in the synthesis of pesticides, lubricants, and other monomers, e.g., 1,4-butanediol.

In the report “Top Value Added Chemicals from Biomass,” the use of glutamic acid and aspartic acid as a building block is reported (Werpy and Petersen 2004). Here, aspartic acid, from the enzymatic conversion of ammonia and fumaric acid (from fermentation), is used as a raw material for a number of chemicals such as 2-amino-1,4-butanediol and amine tetrahydrofuran, which could act as amino analogues of the equivalent C4 compounds.

### Glutamine and glutamic acid

The hydrolysis of amide functionality of glutamine results in the formation of glutamic acid. Just as other α-amino carboxylic acids, glutamic acid can undergo decarboxylation resulting in the formation of γ-aminobutyric acid (GABA). GABA can be found in plants and animal tissues and is formed in the metabolism of l-glutamic acid. Due to its interactions with brain transmitters, GABA-based products for use as sedatives and muscle relaxants have been developed.

The National Renewable Energy Laboratory, Pacific Northwest National Laboratory, and US Department of Energy Office of Energy Efficiency and Renewable Energy report the use of glutamic acid, produced from fermentation, as a building block, where it could be used as a raw material for a number of chemical products such as 5-amino-1-butanol and glutaric acid, which have a commercial application (Werpy and Petersen 2004).

### Glycine

Patients that are fed glycine have similar amounts of oxalates in the urine when compared with patients with hyperoxaluria (elevated oxalates in the urine; Crawhall et al. 1959). The transformation has also been described using chemical methods. When glycine is electrolyzed in nitric acid, it was found that oxalic acid was formed (Takayama 1941). Use of alanine also resulted in the formation of oxalic acid. It is unknown in which volumes are currently prepared; however, it can be said that it is made by a number of producers such as Bayer AG and Ciba Specialty Chemicals and utilized in areas such as a bleaching agent in the textile and pulp industries and wastewater treatment.

### Leucine and isoleucine

**Fig. 1:**
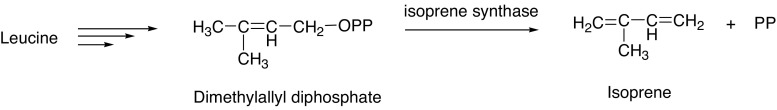
The synthesis of isoprene from leucine

Limited information about the chemical conversions of leucine apart from the (thermal) decomposition to 3-methyl-butylamine have been described. However, interesting biochemical conversion has been reported. The mevalonate pathway in the leucine (and also isoleucine and valine) degradation pathway, leads to the formation of the isoprene precursor, dimethylallyl pyrophosphate (DMAPP; Kegg database, http://www.genome.jp/kegg). For the conversion of DMAPP to isoprene, the enzyme isoprene synthase is required. Extracts from the leaves of *Quercus petraea* were found to contain isoprene synthase. Enzyme activity was found to be dependent on the presence of bivalent cations and temperature. Respectively, Mg^2+^ and 35°C were found to be most favorable (Steinbrecher and Lehning 1996). Other sources of isoprene synthase, such as leaves from *Populus termuloides*, have also been identified (Silver and Fall 1991). It has also been isolated from *Populus alba* (poplar) and the gene encoding isoprene synthase activity isolated and expressed in *Escherichia coli*. The recombinant protein led to a rate of isoprene formation as high as 7.7 nmol related to 1-mg cell protein, after 14 h of culture incubation (Miller et al. 2001). A general scheme for the synthesis of isoprene from leucine is given in Fig. 1.

### Lysine, ornithine, and arginine

Perhaps one of the most well known (and smelliest!) transformations of amino acids are the conversion of lysine and ornithine in decomposing flesh to 1,5-pentanediamine and 1,4-butanediamine known, respectively, as cadaverine and putrescine. Here, the amino acids undergo decarboxylation using the decarboxylase enzymes. In one report, the authors have shown that 1,4-butanediamine can be produced by a microorganism that has increased ornithine decarboxylase and simultaneous *N*-acetylglutamate activity (Eppelmann et al. 2006). Arginine can undergo hydrolysis resulting in the formation of ornithine and urea. This process forms part of the urea cycle in nature and may be carried out utilizing the enzyme arginase (Bach 1939; Albanese et al. 1945) or by chemical means such as montmorillonite (Ikeda and Yasunaga 1984). The resultant ornithine can undergo further reaction as described previously. From these diamine products, only the application of 1,4-butanediamine is known, where it is used as the comonomer, together with adipic acid, in the production of nylon-4,6 (Stanyl®). However, current production uses propylene, ammonia, and hydrocyanic acid and not amino acids as raw materials.

**Fig. 2:**
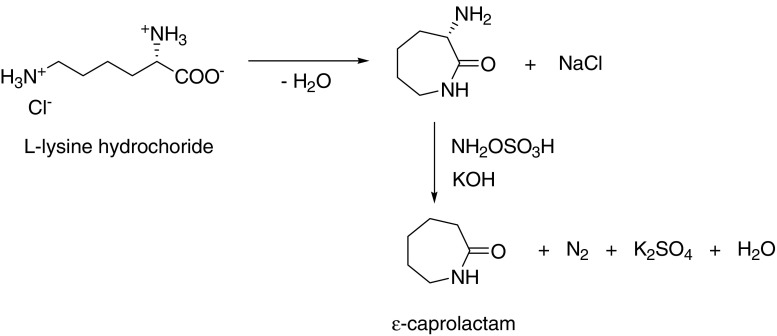
The synthesis of ɛ-caprolactam from lysine

In a patent by Frost (2005), the use of lysine to prepare ɛ-caprolactam is reported. Here, the lysine is derived by fermentation. The lysine (salt), upon heating, cyclises to form the lactam α-amino-ɛ-caprolactam; this is followed by deamination using hydroxylamine-*o*-sulfonic acid. This reaction is described in Fig. 2.

Literature describing the synthesis of intermediates for ɛ-caprolactam, such as the use of (biotechnologically derived) lysine to form 6-aminohex-2-enoic acid (Houben-Weyl 2002) as well as the biochemical production of 6-aminohex-2-enoic acid (Raemakers-Franken et al. 2005) and its conversion to 6-amino caproic acid, have been reported. ɛ-Caprolactam (used in nylon-6 production) has been widely described (Weissermel and Arpe 1993) and generally involves the following steps: synthesis of cyclohexanone, transformation of cyclohexanone to the corresponding oxime, and Beckmann rearrangement of the oxime to ɛ-caprolactam. In these cases, although amino acids are used as a precursor, these amino acids are generated from carbohydrates via fermentation.

### Phenylalanine

Using the enzyme phenylalanine ammonia lyase (PAL, E.C. 4.3.1.5), phenylalanine can be deaminated yielding cinnamic acid. PAL has been extensively studied, together with the reverse reaction of phenylalanine production from cinnamic acid (Camm and Towers 1973; Nijkamp et al. 2005). PAL has been found in various yeasts, and it has been reported that *Rhodotorula glutinis* utilized l-phenylalanine as sole C and N source and accumulated a large amount of *trans*-cinnamic acid, which could be isolated after extraction from the broth (Ogata et al. 1966). In another method, cinnamic acid production using recombinant *E. coli* strains under alkaline conditions yielded about 15% cinnamic acid (Ben-Bassat et al. 2005).

**Fig. 3:**
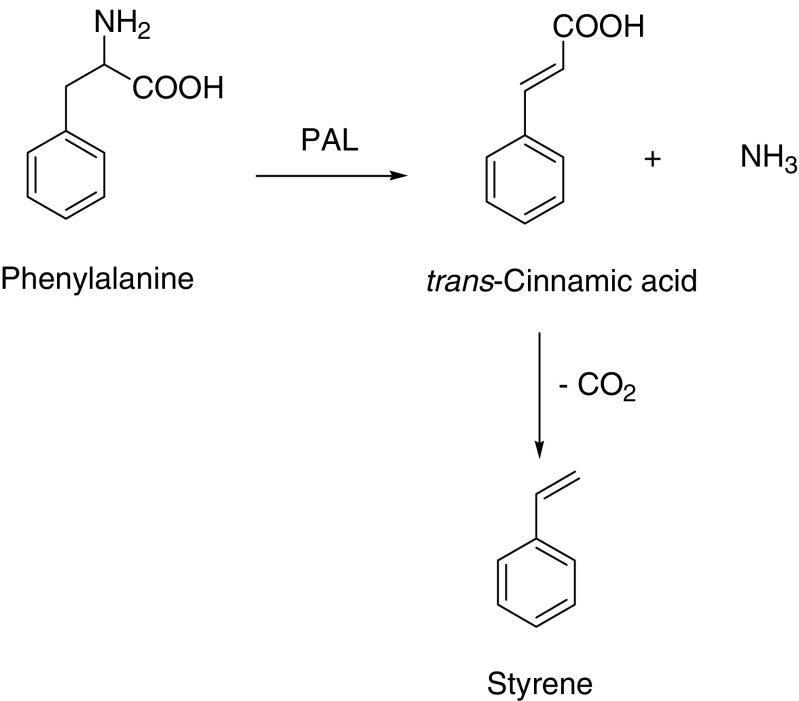
The production of styrene from phenylalanine

The chemical and biocatalytic conversion of cinnamic acid to styrene by decarboxylation has been described, see Fig. 3 for reaction scheme. A study where cinnamic acid was heated to 200–270°C in the presence of a CuSO_4_ catalyst yielded 80–90% styrene (Dahlig 1955). Decarboxylation of *p*-hydroxy-cinnamic acid using a triethylamine catalyzed microwave reaction yielded about 60% of the corresponding decarboxylated compound, while cinnamic acid was found not to react under these conditions (Nomura et al. 2005). Cultures of the yeast *Cryptococcus elinovii* grown on cinnamic acid resulted in partial conversion to styrene, where yields were limited to about 50%, as styrene was toxic to the cells. Using cell-free extracts of the same culture, conversion of cinnamic acid to styrene was also demonstrated (Middelhoven and Sollewijn Gelpke 1995). Plant cell cultures were also employed to decarboxylate cinnamic acid and derivatives. However, using cell cultures from *Camellia sinensis*, cinnamic acid decarboxylation only resulted in low conversion yields (10%) (Takemoto and Achiwa 2001). The major industrial synthesis of styrene utilizes the catalyzed (oxy)dehydrogenation of ethylbenzene. Styrene is exclusively used in the production of poly(styrene).

### Proline

Proline may be produced in microorganisms and plants from glutamate and ornithine. Plants under stress conditions produce proline by cells in response to changes in osmotic pressure. Under some conditions, accumulation can be very high (up to about 80% of the total cellular amino acid content). It has been reported that proline can undergo transformation to γ-butyrolactam (pyrrolidone) by oxidative decarboxylation, via the imine, in the presence of iodobenzene (Ochiai et al. 1988). γ-Butyrolactam is manufactured conventionally by the reaction of butyrolactone (derived from 1,4-butanediol) with ammonia and is used as a precursor for the manufacture of *N*-vinylpyrrolidone used to manufacture poly(vinylpyrrolidone). Poly(vinylpyrrolidone) is a specialty polymer with applications as binders and blood plasma substitutes in the cosmetic and medical sectors and has a price of about €15,000 per tonne.

Other reactions of proline have also been reported such as the formation of pyrrolidine. γ-Radiolysis of an oxygenated aqueous solution of proline showed formation of the hydroxyproline with further irradiation leading to destruction of the pyrrolidine ring (Kopoldova and Voracek-Hubsch 1975). A synthetic procedure involving the conversion of proline to pyrrolidine has also been described (Suyama and Kanao 1965). Here, a number of amino acids were examined when heated in tetralin and cyclohexanol, and it was found that decarboxylation in the case of proline leads to the formation of pyrrolidine. Other decarboxylation studies have also been performed (Dose 1957). Industrial synthesis of pyrrolidine for use in pharmaceuticals is carried out.

### Serine

Serine, just like many α-amino carboxylic acids, can undergo α-decarboxylation. Here, the product of the reaction is ethanolamine (Suyama and Kanao 1965; Elabbadi et al. 1997; Xu et al. 1994). The established biochemical pathways from serine to ethanolamine are indirect and involve decarboxylation of phosphatidylserine, but it was reported that plants also can directly decarboxylate serine (Rontein et al. 2001). This reaction is catalyzed by a pyridoxal 5′-phosphate-dependent l-serine decarboxylase found in soluble extracts from leaves of diverse plant species and could be suitable for a bio-industrial process. Ethanolamine is an industrial product used as an intermediate in the herbicide, textile, metal, detergent, plastics, and personal care products industries with a production volume running into several hundreds of kilotonnes per annum.

### Threonine

As is the case for many of the amino acids (thermo-)chemical decarboxylation of threonine has been extensively reported in the open literature. Studies have looked into the oxidative decarboxylation with periodic acid (Sprinson and Chargaff 1946) and thermal decarboxylation in the presence of ketones (Dose 1957; Chatelus 1964) resulting in the formation of 1-amino-2-hydroxypropane (isopropanolamine) in significant quantities. Isopropanolamine is currently produced in industry by the reaction of propylene oxide and ammonia and used in areas such as the production of surfactants, pigments, lubricants, and foam products. Isopropanolamine can also undergo transformation by gas phase dehydration over an oxide catalyst resulting in the formation of allylamine (Shinkichi et al. 1991). Allylamine is used in antifungal preparations, where conventional industrial production is carried out by reaction of allyl chloride with ammonia.

A number of early studies describe the mechanistic process for deamination of threonine using bacteria such as *E. coli* (Wood and Gunsalus 1949) and *Psedudomonas pyocyanea* (Chargaff and Sprinson 1943) resulting in the formation of 2-oxobutyrate. Conversion of threonine and 2-oxobutyrate by *Fusobacterium* species lead to the formation of 2-hydroxybutyrate and propionate. In some cases, equimolar amounts of propionate (propanoic acid) were generated from threonine (Carlier et al. 1997). Propanoic acid is commercially available for application in antifungal preparations, as intermediate in pesticides and pharmaceuticals and is prepared by the air oxidation of propionaldehyde. However, it is unlikely that the production of propanoic acid from amino acids is competitive, as fermentation technology for its production is advanced.

### Tryptophan

The oxidation of tryptophan can yield a variety of species. This includes the formation of catechol, which may be further oxidized to muconic acid. This latter product was seen to form when purified enzymes were utilized (Suda et al. 1951). It has also been found that accumulation of catechol (and 2,3-dihydroxybenzoic acid) takes place in the culture filtrates of *Aspergillus niger* grown in the presence of l-tryptophan (Rao et al. 1967). When l-tryptophan was used to grow *Bacillus megaterium*, catechol was also formed as an intermediate in the degradation process (Bouknight and Sadoff 1975). Catechol is used in the preparation of photo and printing chemicals.

### Tyrosine

It has previously been discussed that the use of PAL can be used in the deamination of phenylalanine yielding cinnamic acid. In the same report, the use of tyrosine ammonia lyase in conjunction with the deamination of tyrosine to *p*-hydroxy cinnamic acid is described (Ben-Bassat et al. 2005). Where recombinant *E. coli* strains were employed at alkaline pH, better results for the biosynthesis of *p*-hydroxy cinnamic acid compared to cinnamic acid from phenylalanine using the same microorganisms were obtained. PAL can also be utilized in the deamination of tyrosine to *p*-hydroxy cinnamic acid (coumaric acid; Shen and Abel 1985) with some studies focusing on activity of the enzymatic reaction and consequences on plant growth (Walton 1968). The implementation of PAL to make variations of cinnamic acid and *p*-hydroxy cinnamic acid from phenyl alanine and tyrosine derivatives, respectively, has also been studied (Tauchida et al. 1975). The formation of styrene from cinnamic acid by decarboxylation has been carried out by enzymes or traditional chemical methods. In much the same way, *p*-hydroxy cinnamic acid can be converted to *p*-hydroxy styrene. As described earlier, the formation of the *p*-hydroxy styrene was found to form preferentially using microwave-assisted decarboxylation (Nomura et al. 2005). Epiphytic bacteria, *Klebsiella oxytoca* and *Erwinia ureovora* of *Polymnia sonchifolia* leaves, have been used in the biocatalytic decarboxylation of hydroxycinnamic acid to hydroxystyrene, where it was used as a possible detoxification of hydroxycinnamic acid of plants. Hydroxystyrene, also known as 4-vinylphenol, is used as the monomer for the production of poly(4-vinylphenol) (PVP), a polymer structurally similar to polystyrene. PVP is used in the electronics industry as a dielectric layer in transistors applied in liquid crystal displays for example.

### Valine

**Fig. 4:**
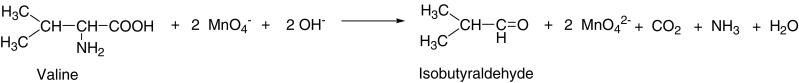
Formation of isobutyraldehyde from valine

Valine can undergo oxidative deamination and decarboxylation resulting in the formation of isobutyraldehyde. Here, valine was reacted with two equivalents of KMnO_4_ in aqueous alkaline solution to form isobutyraldehyde. Although kinetic and mechanistic studies were performed, no preparative synthesis was carried out (Abdulazizkhan et al. 2000). Figure 4 illustrates oxidative deamination and decarboxylation using a chromium (III) catalyzed alkaline manganate solution (Kulkani et al. 2003). Ag(I) catalyzed acidic solutions have also been reported (Chourey et al. 1998).

The enzymatic production of isobutyraldehyde from valine in the presence of polyphenol oxidase and catechin was reported (Motoda 1979). Aldehydes with one carbon atom less than the amino acid were formed by interaction of amino acids and polyphenols catalyzed by polyphenol oxidase. This reaction was also observed in extracts of tea, cocoa, and coffee. Use of polyphenol oxidase, purified from cultures of *Alternaria tenuis*, showed that the rate of aldehyde formation was proportional to the amount of enzyme and amino acid and catechol concentrations. The proposed mechanism involves two steps: enzymatic oxidation of the catechols to the corresponding quinones, followed by quinone-catalyzed Strecker degradation of the amino acid to yield the aldehyde, ammonia, and carbon dioxide. Strecker degradation is well known and important for food aroma evolution. Oxidative deamination of d-valine catalyzed by cell-free extracts from cultures of *Pseudomonas aeruginosa* produced 2-oxoisovalerate, although the corresponding l-amino acid was not oxidized using the same enzyme preparation (Norton and Sokatch 1966). However, oxidation of d,l-valine by d-amino acid oxidase from hog kidney yielded 2-oxoisovalerate with 75% yield (Meister 1952). Further chemical or enzymatic decarboxylation of 2-oxoisovaleric acid yields isobutyraldehyde.

## New approaches in the use of biomass

**Fig. 5:**
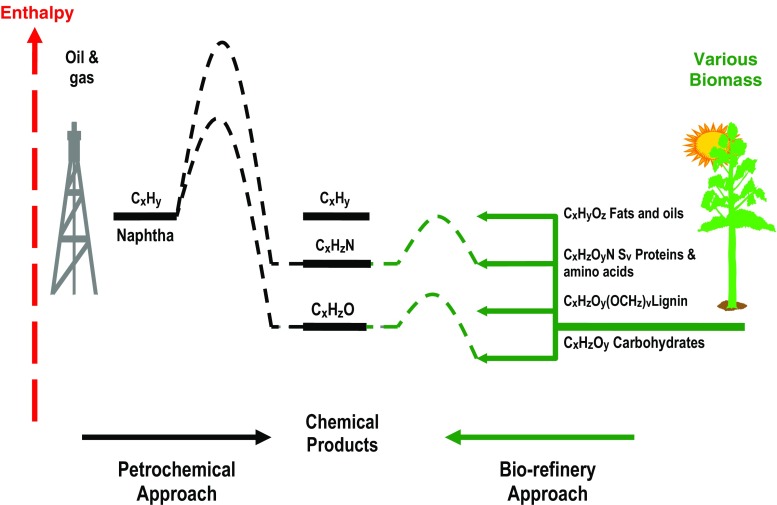
The use of biomass as a more energy-efficient raw material

**Fig. 6:**
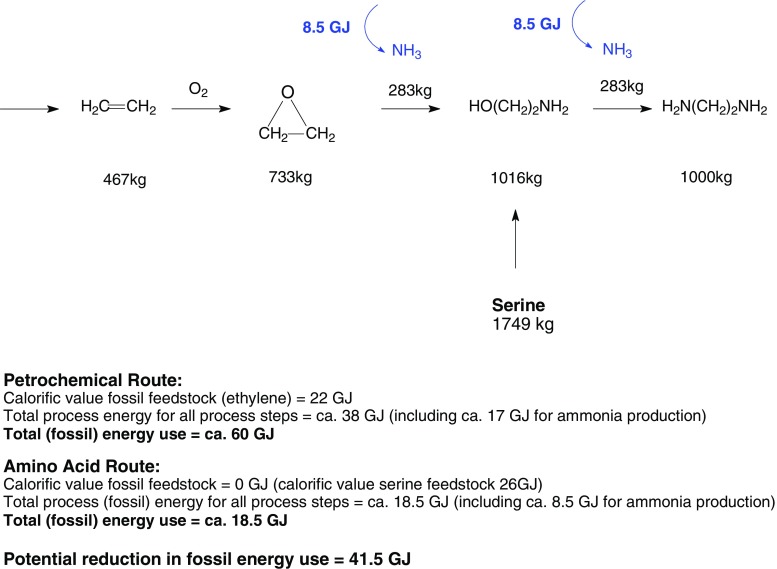
Bio-based vs petrochemical production of 1,2-ethanediamine

Allied to the dwindling fossil feedstocks at every increasing prices, the conversion of crude oil products such as the primary products (ethylene, propylene, etc.) to either materials or other (functional) chemicals requires the use of co-reagents, such as chlorine and ammonia, and various process steps to introduce functionality such as -NH_2_ into the simple structures of the primary products (Brown 2003; Boeriu et al. 2005). Conversely, many products formed in plants often contain this type of functionality. Therefore, it is attractive to exploit this to bypass the use, and preparation, of co-reagents as well as eliminating various process steps by utilizing suitable biomass-based precursors for the production of chemicals. If one considers the enthalpy changes involved the conversion of naphtha from crude oil to chemical products, naphtha has a calorific value of about 45 GJ per tonne, and requires the use of additional (process) energy in the form of heat and electricity to produce a product with a significantly lower calorific value compared to the original fossil raw material, see Fig. 5. On the other hand, components of biomass have calorific values and chemical structure/functionality that, such as amino acids, have a calorific value of about 20–30 GJ per tonne and chemical structures that can be readily converted to amines (of similar calorific values) in fewer process steps and thus with a more limited input of (process) energy, see the following case study and Fig. 6. Amines and diamines have a variety of applications in the chemical and plastics industries. These include chemical intermediates for pharmaceuticals and dyes and monomers for engineering plastics such as nylons.

### Case study: 1,2-ethanediamine synthesis—petrochemical vs amino acid route

In the case of diamines, 1,2-ethanediamine is produced from ethylene by various routes: oxidation of ethylene to the epoxide followed by amination to ethanolamine and then to 1,2-ethanediamine (a new route uses ethane that is oxidized directly to the epoxide). A number of producers use a route via the chlorination of ethylene followed by substitution of the chlorine with ammonia. Production of 1,4-butanediamine starts with propylene and uses ammonia and hydrocyanic acid. Starting from the amino acids serine and arginine, respectively, these diamine products could also be synthesized using well-described enzymatic and/or chemical conversion steps. Formation of the diamine is obtained by reaction of ethanolamine, obtained from serine, with ammonia. The current process for the production of 1,2-ethanediamine (via ethylene oxide) utilizes about 60–70 GJ per tonne (assume about 60 GJ per tonne), where about 22 GJ is in the form of the ethylene raw material and, hence, about 38 GJ in the form of various process energies (including about 17 GJ for ammonia production). By utilizing the amino acid serine in the synthesis, various steps and process energies may be eliminated. On a stoichiometric basis, 1,749 kg of serine is required to produce 1,016 kg of ethanolamine. The calorific value of this amount of serine is about 26 GJ (based on a calorific value of 15 GJ/tonne). In the proposed process, about 8.5 GJ for ammonia production, about 5 GJ for the conversion of serine to ethanolamine, and a further about 5 GJ for the transformation of ethanolamine to 1,2-ethanediamine is required (total 18.5 GJ). Thus, the use of 26 GJ of serine saves about 41.5 GJ of fossil energy use (22 GJ ethylene plus about 19.5 GJ of various process energies). This represents an energy saving of about 41.5 GJ of expensive oil and gas resources. In practice, this saving may be lower, as the production of serine from biomass has not been taken into account. It is expected that the conversion of serine to ethanolamine would require limited energy if carried out using enzymatic technology instead of the chemical process described here, where a process energy of about 5 GJ is assumed (Rabou et al. 2006). If the amino acids would have been used to make electricity instead, by burning this biomass in a power plant, then only the caloric value of these products would have been obtained, being around 20–30 GJ/tonne. This is in competition with 20–30 GJ of inexpensive coal resources.

### Considerations in the use of amino acids as feedstocks

The potential use of amino acids to prepare bulk chemicals is not only limited to formation of simple amines and/or diamines. Indeed, reactions of amino acids, as illustrated earlier, can lead to the formation of a number of chemicals such as ɛ-caprolactam and styrene, which are prepared industrially on a “bulk” scale from petrochemical feedstocks. However, the general focus of these studies relies on the underlying chemistry and/or microbiology and not necessarily the potential of the amino acid as a feedstock for industrial bulk chemical production.

In the strategy of the use of amino acids in the chemical industry (Sanders et al. 2007), several issues should be addressed:

- How is it possible to obtain sufficient volumes of amino acids?
- How is it possible to obtain the correct (single) amino acid (at the correct purity) for the process route in question?
- Is it possible to obtain amino acid feed stocks at the correct feedstock price?
- What technology and infrastructure is required to utilize amino acid feedstocks for chemical production?
- What is the potential impact of using amino acid feedstocks?

#### How is it possible to obtain sufficient volumes of amino acids?

In this study, one must consider nontraditional sources of amino acids. Conventionally, (most) amino acids are obtained from fermentation routes using carbohydrates and nitrogen sources. However, these processes do not offer the possibilities to reduce co-reagents such as ammonia (and/or nitrates), as these are utilized in the amino acid production process itself. As well as this, the production volumes are low and price per tonne is prohibitive for a great number of the amino acids for their application as raw materials for the (bulk) chemical industry. Thus, it is considered that amino acids obtained from inexpensive proteins should be utilized. For example, one can think of Dried Distillers Grain and Solubles (DDGS) obtained from (bio-)ethanol production. Here, the price (source: PERP Report Ethanol 04/058) has drifted from $140 to $90 per short tonne between 1980 and 2000.

Considering that an assumed volume of 10% of the total transportation market in 2020 will consist of biofuels, an additional 100 million tonnes of protein will be produced. Thus, each of the amino acids would become available at a volume of about 5 million tonnes per annum. If one considers the production of a bulk chemical such as acrylic acid (globally 1.5–2 million tonnes per annum), then sufficient amounts of the required amino acid should be available to allow a large proportion of the production to be from bio-based feedstocks.

As well as utilizing waste protein sources, other strategies at isolating amino acids are being developed such as the production of non-ribosomal peptides using inexpensive agro-waste materials. This offers the advantage that only selected amino acids will be included in the peptide, leading to advantages in downstream processing of obtaining amino acids. An example of this is the production of cyanophycin, which is produced using cyanobacteria and consists of a backbone of poly(aspartic acid) with an arginine molecule attached to each aspartic acid unit. A number of recombinant bacteria have now been constructed (Voss et al. 2004) that acquired a cyanophycin synthetase A gene and were able to produce considerable amounts of cyanophycin (up to 40% per cell dry weight). Jointly, Wageningen and Münster Universities have shown that Protamylasse™, a waste stream from the potato starch industry (about 100,000 tonnes per year) that contains large amounts of amino acids, is a very promising substrate for the production of cyanophycin (Elbahloul et al. 2005a,b, 2006; Mooibroek et al. 2006).

#### How is it possible to obtain the correct (single) amino acid (at the correct purity) for the process route in question?

Conventional isolation of amino acids requires some sort of separation, isolation, or purification technique. In some cases, where simple mixtures are obtained, reactive extraction can be carried out where isolation of amino acids with polar side groups (e.g., serine) vs nonpolar side groups (e.g., leucine) or basic amino acids (e.g., lysine) vs acidic amino acids (e.g., glutamic acid) may be achieved. However, where a large number and complex mixture of amino acids are generated, the use of chromatographic techniques is required to obtain specific amino acids at a required purity. Ion exchange chromatography is generally expensive, requiring large amounts of chemical to regenerate the columns as well as producing large amount of by- or waste products. Although using these techniques, the correct (single) amino acid feedstock may be obtained, to address issues such as waste management, scale of operation, and investment, in other words ecology and economy, it is important that developments in the separation of amino acids from (complex mixtures) be investigated.

As discussed above, the production of specific (and insoluble) non-ribosomal peptides, as in the case of cyanophycin, offers the opportunity to isolate specific amino acids from a complex mixture and aid downstream processing.

#### Is it possible to obtain amino acid feedstocks at the correct feedstock price?

Waste proteins, such as those obtained from biofuel production such as DDGS from bioethanol production, and rape and soya cake from biodiesel production are anticipated to cost around €100–200 per tonne of protein. Given this value, there should be enough economic room for the protein hydrolysis and amino acid purification.

#### What technology and infrastructure is required to utilize amino acid feedstocks for chemical production?

Given the large volumes of proteins (and amino acids) anticipated to be generated for potential conversion, ports such as the Port of Rotterdam could be an attractive place to obtain these (waste) proteins and convert them to chemicals due to their current activities in biomass import, biorefining, biofuels, and large and integrated chemical complexes.

#### What is the potential impact of using amino acid feedstocks?

Assume that all the proteins/amino acids (about 100 million tonnes, at a calorific value of about 20–30 GJ per tonne) could be used as raw materials in the chemical industry, this would equate to the replacement of about 2,000–3,000 PJ of fossil resources (in the broadest terms). Should the use of the amino acids also lead to advantage in circumventing process steps and the production and use of co-reagents (as seen in Fig. 6 and case study), then there is a potential for further reduction in the use of fossil energy not only as feedstock but also that used in the form of various process energies including the production of co-reagents. This should also lead to major reductions in capital costs.

## References

[CR1] Abdulazizkhan LH, Kembhavi MR, Nandibevoor ST (2000). Kinetic and mechanistic study of the oxidative deamination and decarboxylation of l-valine by alkaline permanganate. Monatsh Chem.

[CR2] Albanese AA, Irby V, Frankston JE (1945). The utilization of d-amino acids by man. III. Arginine. J Biol Chem.

[CR3] Arisseto AP, Toledo MCF (2006). Acrylamide in foods: a review. Braz J Food Technol.

[CR4] Bach SJ (1939). The mechanism of urea formation. Biochem J.

[CR5] Ben-Bassat A, Sariaslani FS, Huang LL, Patnaik R, Lowe DJ (2005) Methods for the preparation of *para*-hydroxycinnamic acid and cinnamic acid at alkaline pH. WO20050260724 A1

[CR6] Biebl H, Menzel K, Zeng A-P, Deckwer W-D (1999). Microbial production of 1,3-propanediol. Appl Microbiol Biotechnol.

[CR7] Boeriu CG, van Dam JEG, Sanders JPM, Lens P, Westermann P, Haberbauer M, Moreno A (2005). Biomass valorisation for sustainable development. Biofuels for fuel cells: renewable energy from biomass fermentation (integrated environmental technology series).

[CR8] Bouknight RR, Sadoff HL (1975). Tryptophan catabolism in *Bacillus megaterium*. J Bacteriol.

[CR9] Brown RC (2003). Biorenewable resources: engineering new products from agriculture.

[CR10] Camm EL, Towers GHN (1973). Phenylalanine ammonia lyase. Phytochemistry.

[CR11] Carlier JP, Henry C, Lorin V, Rouffignat K (1997). Conversion of dl-threonine, d-threonine and 2-oxobutyrate into propionate and 2-hydroxybutyrate by *Fusobacterium* species. Lett Appl Microbiol.

[CR12] Chargaff E, Sprinson DB (1943). Studies on the mechanism of deamination of serine and threonine in biological systems. J Biol Chem.

[CR13] Chatelus G (1964). Thermal decarboxylation of α-amino acids. Bull Soc Chim Fr.

[CR14] Chem Systems Reports, Process Evaluation/Research Planning program report (2002) Acrylamide (01/02S10). Chem Systems Reports

[CR15] Chemical Week (2005) ADM plans polyols unit using renewable feedstock. Chem Week (30 Nov/7 Dec) p 23

[CR16] Chemie Magazine (2006) Hoger rendement door lagere energiekosten: methanor verder met biomethanol. Chem Mag (October) http://www.vnci.nl/actueel/chemiemagazine/default.asp

[CR17] Chourey VR, Pandey S, Shastry LV, Shastry VR (1998). Kinetics of silver (I) ion catalyzed deamination and decarboxylation of d,l-valine by acidic permanganate. Chim Acta Turc.

[CR18] Crawhall JC, De Mowbray RR, Scowen EF, Watts RWE (1959). Conversion of glycine to oxalate in a normal subject. Lancet.

[CR19] Crocomo OJ, Fowden L (1970). Amino acid decarboxylases of higher plants: formation of ethylamine. Phytochemistry.

[CR20] Dahlig W (1955). Styrene from cinnamic acid. Przemysl Chemiczny.

[CR21] Dose K (1957). A new method for the preparation of amines from α-amino carboxylic acids. Chem Ber.

[CR22] Elabbadi N, Ancelin ML, Vial HJ (1997). Phospholipid metabolism of serine in Plasmodium-infected erythrocytes involves phosphatidylserine and direct serine decarboxylation. Biochem J.

[CR23] Elbahloul Y, Frey K, Sanders J, Steinbüchel A (2005). Protamylasse, a residual compound of industrial starch production, provides a suitable medium for large-scale cyanophycin production. Appl Environ Microbiol.

[CR24] Elbahloul Y, Mooibroek H, Sanders J, Steinbüchel A (2005b) Protamylasse, a residual compound suitable for large scale cyanophycin production. Renewable resources and biorefineries conference, September 19–21 2005, Ghent, Belgium

[CR25] Elbahloul Y, Sanders JPM, Scott EL, Mooibroek H, Obst M, Steinbüchel A (2006) Cyanophycin production from nitrogen containing chemicals obtained from biomass. WO2006093411

[CR26] Eppelmann K, Nossin PMM, Raeven LJRM, Kremer SM, Wubbolts MG (2006) Biochemical Synthesis of 1,4-butanediamine. WO2006005603

[CR27] Frost JW (2005) Synthesis of caprolactam from lysine. WO2005123669

[CR28] Goekman V, Senyuva HZ (2006). A simplified approach for the kinetic characterisation of acrylamide formation in fructose–asparagine model system. Food Addit Contam.

[CR29] Houben-Weyl (2002). Methods of organic chemistry, vol E22a.

[CR30] Ikeda T, Yasunaga TJ (1984). Kinetic behaviour of l-arginine in the interlamellar layer of montmorillonite in aqueous suspension. J Phys Chem.

[CR31] Kopoldova J, Voracek-Hubsch M (1975). Gamma radiolysis of aqueous solution of proline. Zeitschrift fuer Naturforschung. J Biosci.

[CR32] Kulkani RM, Bilehal DC, Nandibevoor ST (2003). Deamination and decarboxylation in the chromium (III)-catalyzed oxidation of l-valine by alkaline permanganate and analysis of chromium (III) in microscopic amounts by a kinetic method. Transit Met Chem.

[CR33] Lange J-P (2001). Fuels and chemicals manufacturing guidelines for understanding and minimizing the production costs. CaTTech.

[CR34] Meister A (1952). Enzymatic preparation of α-keto acids. J Biol Chem.

[CR35] Middelhoven WJ, Sollewijn Gelpke MD (1995). Partial conversion of cinnamic acid into styrene by growing cultures and cell-free extracts of the yeast *Cryptococcus elinovii*. Antonie van Leeuwenhoek.

[CR36] Miller B, Oschinski C, Zimmer W (2001). First isolation of an isoprene synthase gene from poplar and successful expression of the gene in *Escherichia coli*. Planta.

[CR37] Mooibroek H, Franssen H, Scott E, Sanders J, Steinbüchel A (2006) BASF symposium on bioinspired materials for the chemical Industry. ISIS, August 7–9 2006, Strasbourg, France

[CR38] Motoda S (1979). Enzymic production of aldehydes from amino acids by polyphenol oxidase. J Ferment Technol.

[CR39] Nijkamp K, van Luijk N, de Bont JAM (2005). The solvent-tolerant *Pseudomonas putida* S12 as host for the production of cinnamic acid from glucose. Appl Microbiol Biotechnol.

[CR40] Nomura E, Hosoda A, Mori H, Taniguchi H (2005). Rapid base-catalyzed decarboxylation and amide-forming reaction of substituted cinnamic acids via microwave heating. Green Chem.

[CR41] Norton JE, Sokatch JR (1966). Oxidation of d- and l-valine by enzymes of *Pseudomonas aeruginosa*. J Bacteriol.

[CR42] Ochiai M, Inenaga M, Nago Y, Moriarty RM, Vaid RK, Duncan MP (1988). Oxidative decarboxylation of cyclic amino acids and dehydrogenation of cyclic secondary amines with iodosobenzene. Tetrahedron Lett.

[CR43] Ogata K, Uchiyama K, Yamada H (1966). Microbial formation of cinnamic acid from phenylalanine. Agric Biol Chem (Tokyo).

[CR44] Rabou LPLM, Deurwaarder EP, Elbersen HW, Scott EL (2006). Biomassa in de Nederlandse enegiehuishouding in 2030. Report for the platform groene grondstoffen.

[CR45] Raemakers-Franken PC, Nossin PMM, Brandts PM, Wubbolts MG, Peeters WPH, Ernste S, Wildeman d SMA, Schuermann M (2005) Biochemical synthesis of 6-amino caproic acid. WO2005068643

[CR46] Rao SPV, Moore K, Towers NGH (1967). Conversion of tryptophan to 2,3-dihydroxybenzoic acid and catechol by *Aspergillus niger*. Biochem Biophys Res Commun.

[CR47] Robert F, Vuataz G, Pollien P, Saucy F, Alonso M-I, Bauwens I, Blank I (2004). Acrylamide formation from asparagine under low moisture reaction conditions. 1. Physical and chemical aspects in crystalline model systems. J Agric Food Chem.

[CR48] Rontein D, Nishida I, Tashiro G, Yoshioka K, Wu W-I, Voelker DR, Basset G, Hanson AD (2001). Plants synthesize ethanolamine by direct decarboxylation of serine using a pyridoxal phosphate enzyme. J Biol Chem.

[CR49] Sanders J, Scott E, Weusthuis R, Mooibroek H (2007). Bio-refinery as the bio-inspired process to bulk chemicals. Macromol Biosci.

[CR50] Sato N, Quitain AT, Kang K, Daimon H, Fujie K (2004). Reaction kinetics of amino acid decomposition in high temperature and pressure water. Ind Eng Chem Res.

[CR51] Shen RS, Abel CW, Bergmeyer HU (1985). l-phenylalanine and l-tyrosine. Methods of enzymatic analysis, vol 8.

[CR52] Shinkichi S, Takayuki S, Hideki N (1991) Process for preparing monoallylamine. EP433959

[CR53] Silver GM, Fall R (1991). Enzymic synthesis of isoprene from dimethylallyl diphosphate in aspen leaf extracts. Plant Physiol.

[CR54] Sohn M, Ho C-T (1995). Ammonia generation during thermal degradation of amino acids. J Agric Food Chem.

[CR55] Solvay Chemicals (2006) Solvay builds new epichlorohydrin plant to meet growing demands with innovative production process. http://www.solvaychemicals.com

[CR56] Sprinson DB, Chargaff E (1946). Oxidative decarboxylations with periodic acid. J Biol Chem.

[CR57] Steinbrecher R, Lehning A (1996). Characterisation of an isoprene synthase from leaves of *Quercus petraea*. Bot Acta.

[CR58] Suda M, Hashimoto K, Matsuoka H, Kamahora T (1951). Further studies on pyrocatecase. J Biochem.

[CR59] Suyama T, Kanao S (1965). Decarboxylation of amino acids IV. Yakugaku Zasshi.

[CR60] Taeymans D, Wood J, Ashby P, Blank I, Studer A, Stadler R, Gonde P, Eijck P, Lalljie S, Lingnert H, Lindblom M, Matissek R, Mueller D, Tallmadge D, O’Brien J, Thompson S, Silvani D, Whitmore T (2004). A review of acrylamide: an industry perspective on research, analysis, formation and control. Food Sci Nutr.

[CR61] Takano Y, Kaneko T, Kobayashi K, Hiroishi D, Ikeda H, Marumo K (2004). Experimental verification of photostability for free and bound amino acids exposed to gamma rays and UV radiation. Earth Planets Space.

[CR62] Takayama Y (1941). Amino acids and their related compounds. XIII. Electrolysis of some amino acids in nitric acid. Nippon Kagaku Kaishi.

[CR63] Takemoto M, Achiwa K (2001). Synthesis of styrenes through the biocatalytic decarboxylation of *trans*-cinnamic acids by plant cell cultures. Chem Pharm Bull.

[CR64] Tauchida T, Kamijo H, Momose H, Hirose Y (1975) Enzymatic production of cinnamic and *p*-coumaric acid derivatives. JP1973122032

[CR65] Voss I, Diniz SC, Aboulmagd E, Steinbüchel A (2004). Identification of the *Anabaena* sp. strain PCC7120 cyanophycin synthetase as suitable enzyme for production of cyanophycin in gram-negative bacteria like *Pseudomonas putida* and *Ralstonia eutropha*. Biomacromolecules.

[CR66] Walton DC (1968). l-phenylalanine ammonia lyase activity during germination of *Phaseolus vulgaris*. Plant Physiol.

[CR67] Weissermel K, Arpe HJ (1993). Industrial organic chemistry.

[CR68] Werpy T, Petersen G (eds) (2004) Top value added chemicals from biomass. PNNL, NREL, EERE (report 8674)

[CR69] Wood WA, Gunsalus IC (1949). Serine and threonine deaminases of *Escherichia coli*: activators for a cell free enzyme. J Biol Chem.

[CR70] Xu Z, Byers DM, Palmer FB, Cook HW (1994). Serine and ethanolamine incorporation into different plasmalogen pools: subcellular analyses of phosphoglyceride synthesis in cultured glioma cells. Neurochem Res.

[CR71] Yaylayan VA, Locas CP, Wnorowski A, O’Brien J (2005). Mechanistic pathways of the formation of acrylamide from different amino acids. Adv Exp Med Biol.

